# NMDA Receptor Dependent Long-term Potentiation in Chronic Pain

**DOI:** 10.1007/s11064-018-2614-8

**Published:** 2018-08-14

**Authors:** Xu-Hui Li, Hui-Hui Miao, Min Zhuo

**Affiliations:** 1 Center for Neuron and Disease, Frontier Institute of Science and Technology, Xi’an Jiaotong University, Xi’an, 710049 Shaanxi China; 2 Department of Physiology, Faculty of Medicine, University of Toronto, Medical Science Building, 1 King’s College Circle, Toronto, ON M5S 1A8 Canada; 3 Department of Physiology, Faculty of Medicine, University of Toronto, Medical Science Building, Room #3328, 1 King’s College Circle, Toronto, ON M5S 1A8 Canada

**Keywords:** NMDA receptor, Long-term potentiation, ACC, IC, Chronic pain, AC1

## Abstract

Since the discovery of NMDA receptor (NMDAR) dependent long-term potentiation (LTP) in the hippocampus, many studies have demonstrated that NMDAR dependent LTP exists throughout central synapses, including those involved in sensory transmission and perception. NMDAR LTP has been reported in spinal cord dorsal horn synapses, anterior cingulate cortex and insular cortex. Behavioral, genetic and pharmacological studies show that inhibiting or reducing NMDAR LTP produced analgesic effects in animal models of chronic pain. Investigation of signalling mechanisms for NMDAR LTP may provide novel targets for future treatment of chronic pain.

## Introduction

Hippocampal long-term potentiation (LTP) is a well investigated form of synaptic plasticity in the central nervous system. In the hippocampus, LTP is an activity-dependent form of synaptic plasticity in which increased synaptic transmission follows brief, high-frequency stimulation of input pathways. Three different major types of glutamate receptors have been found in the hippocampus: *N*-methyl-d-aspartic acid (NMDA), α-amino-3-hydroxy-5-methyl-4-isoxazole-propionic acid (AMPA) and metabotropic glutamate receptors (mGluRs). While AMPA receptors (AMPARs) mediate normal synaptic transmission, activation of NMDA receptors (NMDARs) is required for the induction of LTP. Pre-treatment with the NMDAR antagonist d-2-amino-5-phosphonopentanoic acid (AP5) prevents the induction of LTP. Subsequent pharmacological and genetic studies have confirmed that NMDAR LTP in the hippocampus play important roles in spatial memory of the hippocampus [1]. New mechanism for the synaptic plasticity in the hippocampus has been cumulatively reported (for example, see [2]). In this article that contributes to the special issue for the discovery of NMDAR LTP by Prof. Graham Collingridge, we will review the roles of NMDAR LTP in pain systems, especially in the condition of chronic pain.

## Basic Circuitry of Pain: From Periphery to the Cortex

Glutamate is the fast excitatory transmitter of first sensory synapses. Peripheral noxious stimuli activate nociceptive afferent fibers (A_δ_ and C fibers) and incoming action potentials trigger a release of glutamate in the spinal dorsal horn. In addition, some neuropeptides such as substance P (SP) and neurokinin A (NKA) are also released in the spinal dorsal horn. Glutamate and neuropeptides activate spinal dorsal horn neurons, including those that send projection terminals to supraspinal structures. Neurons in the thalamus play key roles in relaying these ascending inputs to different cortical area. Among them, anterior cingulate cortex (ACC) and insular cortex (IC) are believed to be critical the unpleasantness of pain. As protective responses to noxious stimuli, endogenous pain modulatory systems are also activated. Descending modulatory systems are biphasic, including descending inhibitory and facilitatory systems. LTP has been reported in synapses located in spinal cord dorsal horn and cortex that are related to pain.

## NMDAR LTP in Spinal Dorsal Horn

Glutamate is a major transmitter between primary afferent fibers and spinal dorsal horn neurons, whereas neuropeptides (e.g., SP and NKA) mediate slow excitatory postsynaptic potentials (EPSPs) at synapses between small-myelinated A_δ_ and unmyelinated C fibers and dorsal horn neurons [3–6]. Fast excitatory synaptic transmission is mainly mediated by AMPAR and kainate receptor (KAR) [7]. In some synapses (or called silent synapses), synaptic responses can be mediated by pure NMDARs [4].

Unlike with the hippocampus, studies of spinal LTP are limited by the technical difficulty of spinal cord slices and the complexity of the spinal local neuronal network. Electrophysiological experiments using intracellular or whole-cell patch-clamp recordings from the spinal dorsal horn neurons generate some important findings related to spinal LTP. Strong tetanic stimulation (100 Hz, 1 s for three times at 10 s intervals) of the dorsal root induces long-lasting enhancement of synaptic responses to presynaptic stimulation [8, 9]. The enhancement is relatively long-lasting (ranging from 25 to 90 min) and input specific. Postsynaptic depolarization of dorsal horn neurons is critical for the induction of spinal LTP. Pairing postsynaptic depolarization with synaptic activity also induces long-lasting enhancement of synaptic responses. Interestingly, the level of postsynaptic depolarization may be important in determining whether synaptic transmission will be potentiated or depressed. In some experiments, synaptic depression can also be induced by pairing synaptic activity with modest postsynaptic depolarization. The induction of spinal LTP requires activation of NMDARs and/or the SP (NK1) receptors. Activation of NMDARs in spinal dorsal horn neurons leads to increases in intracellular Ca^2+^ [10]. Pre-treatment of spinal cord slices with an NMDAR antagonist AP5 prevents the induction of LTP. The contribution of neuropeptide SP to spinal LTP may act by enhancing NMDAR mediated currents in spinal dorsal projecting neurons [8]. The intracellular signal pathways of spinal LTP remain to be fully mapped. Evidence from other studies indirectly indicates that several protein kinases may be important for spinal LTP, such as phospholipid-dependent protein kinase C (PKC). Phorbol ester induces long-lasting facilitation of evoked EPSPs or EPSCs (excitatory postsynaptic currents) amplitude to stimulation of presynaptic fibers [11, 12]. One possible mechanism for PKC-dependent spinal LTP is through the recruitment of spinal silent synapses or the insertion of AMPARs. Brain-derived neurotrophic factor (BDNF) can induce NMDAR LTP in spinal dorsal horn via different signaling pathways [13].

## Early Genetic Studies of NMDAR GluN2B (Also Known as NR2B) in Pain-Related Cortex

In addition to the spinal cord, early studies suggest that NMDAR in supraspinal structures may contribute to persistent or chronic pain. Direct evidence for the contribution of NMDARs in forebrains to behavioral nociceptive responses comes from genetic studies of NMDAR GluN2B transgenic mice. Tang et al. generated transgenic mice with forebrain-targeted GluN2B overexpression, and the normal developmental change in NMDAR kinetics was reversed [14]. GluN2B subunit expression was observed extensively throughout the cerebral cortex including the ACC and IC. In both the ACC and IC, GluN2B expression was significantly increased, and NMDAR mediated responses were enhanced [15]. Interestingly, while transgenic mice and wild-type mice were indistinguishable in tests of acute nociception, GluN2B transgenic mice exhibited enhanced behavioral responses after peripheral inflammation. These findings provide the first genetic evidence that forebrain NMDARs play a critical role in chronic pain.

## NMDAR Dependent Postsynaptic LTP (Post-LTP) in the ACC

### Stimulation Protocols

Among sensory-related cortical areas, LTP is well investigated in the ACC. In the ACC slices of adult animals, different stimulation protocols can be used to induce LTP using field recording and whole-cell patch-clamp recording techniques [16, 17]. For field recording from adult rat or mouse ACC slices, glutamatergic synapses in the ACC can undergo LTP in response to theta burst stimulation (TBS), a paradigm more closely to the activity of the ACC neurons. The potentiation lasted for at least 40–120 min [18]. Unlike the hippocampus, strong tetanic stimulation in the ACC did not cause reliable LTP. Whole-cell patch-clamp recordings allow better investigation of synaptic mechanisms for LTP in the ACC [16]. LTP can be induced using three different protocols, including the pairing training protocol, the spike-timing (spike-EPSPs) protocol, and TBS protocol [16, 19]. LTP induced by the pairing protocol is mainly triggered by the activation of NMDARs, but not through L-type voltage-gated calcium channels (L-VGCCs) [16].

### NMDAR Dependent ACC LTP

In the ACC, NMDAR containing GluN2A or GluN2B subunits contribute to most NMDAR currents [16]. Bath application of a NMDAR GluN2A antagonist (NVP-AAM077) and GluN2B antagonist (ifenprodil/Ro compounds) produce almost completely blockade of NMDAR mediated EPSCs. Application of NMDAR GluN2A or GluN2B antagonist reduces ACC LTP, without complete abolishment of LTP. LTP is only abolished after the co-application of both inhibitors [16]. It is noted that LTP induced by spike-timing protocol seems to be more sensitive to NMDAR GluN2B blockade as compared with effects on LTP induced by pairing training protocol [16].

### NMDAR Dependent Calcium Influx

**Fig. 1:**
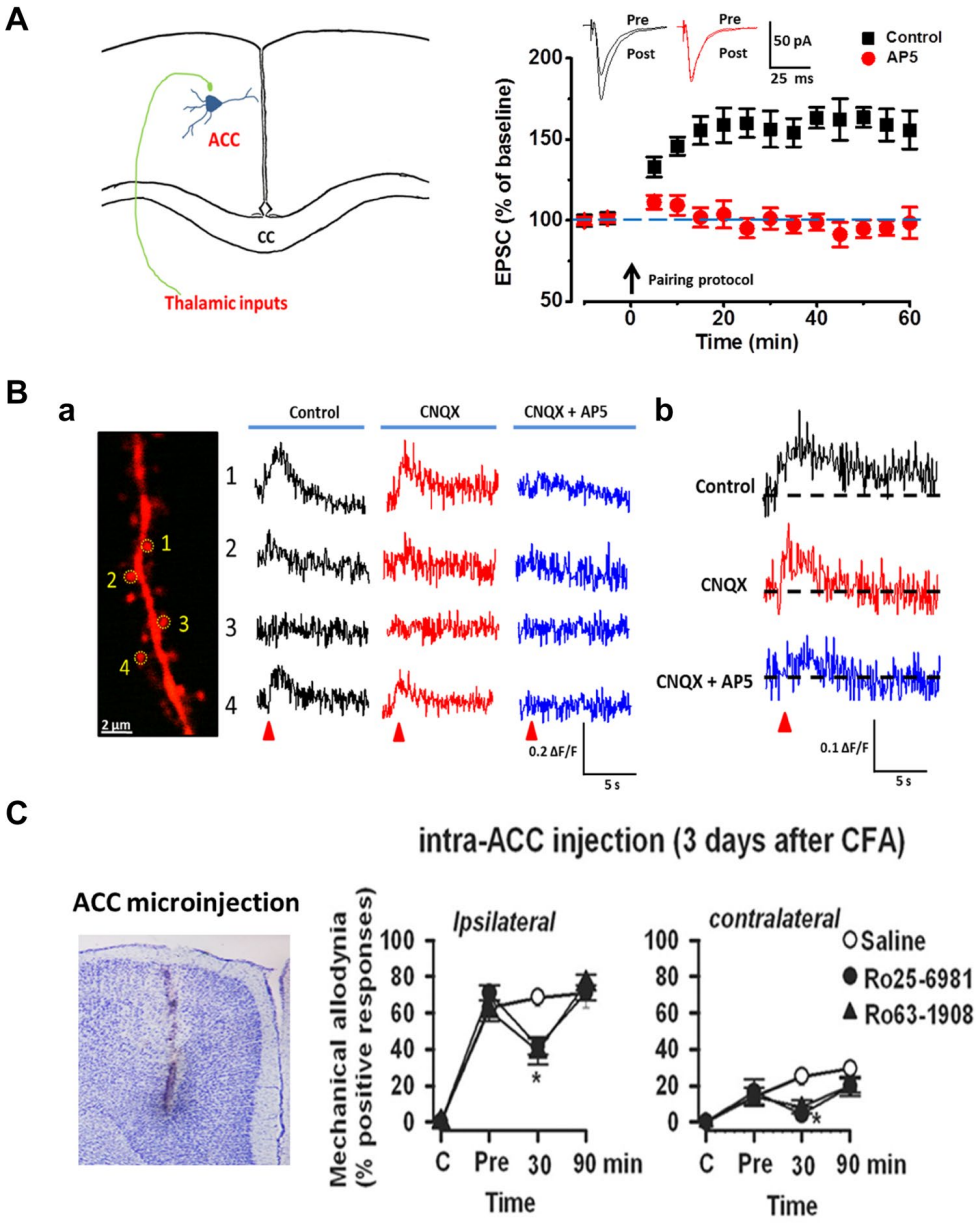
NMDAR dependent LTP in the ACC. **A** Neurons in the ACC receive sensory afferents from the thalamus. Synaptic LTP is believed to be the key cellular mechanism for chronic pain in the ACC. LTP can be blocked by NMDAR antagonist AP5. **B** NMDAR mediated postsynaptic calcium signals in the ACC neurons. Representative calcium transient waveforms and average traces of fluorescence changes (ΔF/F) in responsive spines evoked by puff-application of glutamate in the control artificial cerebro-spinal fluid (ACSF), presence of CNQX (20 µM), and AP5 (50 µM) in the ACC, respectively. **C** ACC microinjection NMDAR antagonists reduced chronic pain. The NR2B receptor antagonists Ro 25-6981 and Ro 63-1908 microinjected into the ACC significantly reduced mechanical allodynia in the CFA injection 3 days mice. Reproduced with permission from Li et al. [19] and Wu et al. [51]

Calcium (Ca^2+^) signaling is critical for the induction of NMDAR LTP [16, 20]. Using the two-photon imaging method, it has been shown that NMDARs contribute to postsynaptic Ca^2+^ signal increases induced by different synaptic stimulation in ACC pyramidal neurons. Furthermore, LTP inducing protocols also triggered postsynaptic Ca^2+^ influx, which were NMDAR dependent [19]. These studies provide the first direct study of Ca^2+^ signals in the ACC and demonstrate that NMDARs play important roles in postsynaptic Ca^2+^ signals (see Fig. 1). As a result of postsynaptic increase of Ca^2+^, Ca^2+^ binds to calmodulin (CaM) and leads to activation of Ca^2+^-stimulated signaling pathways [21]. In support of the role of Ca^2+^ in LTP, postsynaptic injection of 1,2-bis(*o*-aminophenoxy)ethane-*N,N,N*′,*N*′-tetraacetic acid (BAPTA) completely blocked the induction of LTP [16]. Furthermore, a study using electroporation of mutant CaM in the ACC suggests that Ca^2+^ binding sites of CaM are critical for the induction of cingulate LTP [21].

### Downstream Intracellular Signaling Pathways

Cyclic AMP (cAMP) signaling pathways are important signaling pathways in biological systems. Among more than 10 adenylyl cyclase (AC) subunits, AC subtype 1 (AC1) and subtype 8 (AC8) are two AC subtypes that respond positively to Ca^2+^–CaM [22]. As compared with AC8, AC1 is more sensitive to Ca^2+^ increase. In the ACC, AC1 is highly expressed in cingulate neurons located in most layers [23]. AC1 is selective for plastic changes and gene deletion of AC1 does not affect basal glutamate transmission in the ACC. By contrast, LTP induced by TBS or pairing stimulation are abolished in cingulate pyramidal cells [24]. Whole-cell patch-clamp recording also revealed that AC1 activity is required for the induction of LTP in ACC pyramidal cells. By using chemical design and biochemical screening, several selective inhibitors of AC1 have been identified. Consistently, pharmacological inhibition of AC1 in the ACC neurons abolished LTP induced by pairing training [25, 26].

### Expression of ACC LTP

At least four possible mechanisms may contribute to the expression of LTP: (1) presynaptic enhancement of glutamate release; (2) postsynaptic enhancement of glutamate receptor mediated responses; (3) recruitment of previously ‘silent’ synapses or synaptic trafficking or insertion of AMPA receptors; (4) structural changes. Under in vitro brain slice conditions, it appears that the LTP mechanism may depend on the induction protocol in certain cases. Paired-pulse facilitation (PPF) was not altered after the induction of cingulate LTP [16]. However, we do not rule out the possibility of presynaptic changes in the ACC during other physiological/pathological conditions. Among these possibilities, we have recently investigated the roles of AMPAR GluA1 and GluA2/3 using genetic and pharmacological approaches. We found that GluA1 subunit C-terminal peptide analog, Pep1-TGL, blocked the induction of cingulate LTP [27]. Thus, in the ACC, the interaction between the C-terminus of GluA1 and postsynaptic density-95/Discs large/zona occludens-1 (PDZ) domain proteins is required for the induction of LTP. Synaptic delivery of the GluA1 subunit from extrasynaptic sites is the key mechanism underlying synaptic plasticity [28, 29] and GluA1–PDZ interactions are a critical intermediate in this plasticity. Our pharmacological experiments show that the application of philanthotoxin-74 (PhTx) 5 min after paired training reduced to synaptic potentiation, while PhTx had no effect on basal responses. Therefore, we believe that Ca^2+^-permeable GluA2-lacking receptors contribute to the maintenance of LTP and are necessary for subsequent LTP stabilization. These findings indicate that it is likely that selective contribution of AMPARs to cingulate LTP. Recently, we found that the expression of LTP and network LTP was significantly impaired in the GluA1 knock-in mutation mice at the protein kinase A (PKA) phosphorylation site serine 845 (s845A), but not in the CaMKII/PKC phosphorylation site serine 831 (s831A) mice. These results provide strong evidence that PKA phosphorylation of GluA1 is important for the network LTP expression in the ACC [30].

GluA2/3 subunits may continually replace synaptic GluA2/3 subunits in an activity-independent manner that maintains constant synaptic transmission [31–34]. We also examined the role of these peptides in synaptic potentiation in the ACC and found that the GluA2/3–PDZ interaction had no effect on cingulate LTP. We did find that the same interfering peptides inhibited cingulate long-term depression (LTD) [35]. These findings suggest that GluA1 and GluA2/3 play different roles in cingulate LTP vs LTD.

## LTP in the IC

Both the ACC and IC are key cortical areas for pain perception, in addition to other key brain functions [20, 26, 36, 37]. Excitatory synapses in the ACC are highly plastic, and LTP of excitatory transmission can be induced experimentally in brain slice preparation and in vivo conditions by periphery injury. Recent studies characterized LTP in the IC using 64-channel recording system [38]. TBS induced a prolonged potentiation lasting for at least 3 h. The induction of IC LTP is NMDAR dependent, and both NMDAR GluN2A and GluN2B receptors are required for the induction of LTP [38]. Using in vivo recording techniques, it has bene reported that IC LTP induced by tetanic stimulation applied to the basolateral nucleus of the amygdala is blocked by NMDAR antagonist 3-(R-2-carboxypiperazin-4-yl)-propyl-1-phosphonic acid (CPP) in adult rats [39]. Using in vivo optical imaging methods, Mizoguchi et al. has reported that tetanic stimulation caused LTP in the IC of adult rats [40].

IC LTP can also be induced by the LTP pairing protocol using whole-cell patch-clamp recording. We found that, using a pairing training protocol, LTP was induced in pyramidal neurons in the IC slices. NMDAR is important for the induction of LTP, since application of AP5 blocked the induction of LTP. NMDAR GluN2B is important and Ro 25-6981, a selective GluN2B containing NMDAR antagonist, also significantly reduced IC LTP [41]. We also found that postsynaptic calcium is important for the induction of post-LTP, since the postsynaptic application of BAPTA completely blocked the induction of LTP. Ca^2+^-activated AC1 is required for potentiation. By contrast, AC8 is not required. Inhibition of Ca^2+^ permeable AMPA receptors (CP-AMPARs) or protein kinase M zeta (PKMζ) reduced the expression of LTP in the IC [42].

During IC LTP, the paired-pulse ratio is not affected at 1 and 3 h after LTP induction, suggesting that the potentiation is likely not purely expressed presynaptically. Application of 1-naphthyl acetyl spermine (NASPM) (50 µM), a potent CP-AMPARs blocker, reversed the potentiation at 3 h after LTP induction, suggesting postsynaptic recruitment of CP-AMPARs is a necessary step for IC L-LTP expression [43, 44].

AC1 is the major Ca^2+^/calmodulin-stimulated AC isoform among the cAMP signaling pathway [45, 46]. Using knockout mice lacking AC1 (*AC1*^−/−^ mice), we found that the amount of synaptic GluA1 and its phosphorylation at the Ser845 site remained unchanged in the IC after injury. Furthermore, no upregulation of A-kinase anchoring protein (AKAP)79/150, PKA catalytic subunit α (Cα), or PKA regulatory subunit IIβ (RIIβ) were detected from the *AC1*^−/−^mice with nerve ligation. Taken together, these results indicate that AC1 is essential for the translocation of AKAP79/150 and PKA to the synaptic site, and then for the enhancement of synaptic GluA1 in the IC.

## Functional Implications of NMDAR in Chronic Pain

### Spinal Cord

It is well known that NMDARs play important roles in spinal pain processes. For example, responses of sensory dorsal horn neurons to noxious and non-noxious stimuli are enhanced by application of NMDA, indicating that the NMDAR may contribute to both hyperalgesia and allodynia [47]. Activation of NMDARs in the spinal cord by glutamate or NMDA produces nociception or behavioral hyperalgesia and the effects are blocked by an NMDARs antagonist. NMDA excites spinal dorsal horn neurons (including identified spinothalamic tract cells) and enhances their response to peripheral sensory stimuli. Blockage of NMDARs in the spinal cord by different antagonists reduces thermal hyperalgesia, mechanical hyperalgesia/allodynia, and spontaneous nociceptive behavior or autotomy (a behavioral model of neuropathic pain) in rats with different types of persistent pain [47]. NMDAR antagonists applied systemically or intrathecally significantly reduce pain sensation in patients with different kinds of persistent pain [48]. For example, Kristensen et al. reported that intrathecal administration of a NMDAR antagonist relieved pain sensation to mechanical and thermal stimuli in a patient with nerve injury, while the allodynia response, as well as deep pain sensation, were still present [49]. Lagraize et al. found that intrathecal (i.t.) injection the NMDAR channel blocker MK-801 blocked complete Freund’s adjuvant (CFA) induced thermal hyperalgesia [50]. These results suggest that NMDAR dependent synaptic plasticity may actually play an important role in the induction and/or maintenance of persistent pain. Future studies to directly address the relationship between spinal LTP and persistent pain are needed.

### Cortex

Does genetic overexpression of NMDAR GluN2B mimic physiological or pathological conditions? Our study provides the first evidence that the up-regulation of NMDAR GluN2B in the ACC contributes to inflammation-related persistent pain. After persistent inflammation, the expression of NMDAR GluN2B in the ACC was up-regulated, thereby increasing the GluN2B component in NMDAR mediated responses [51]. Consistently, microinjection into the ACC and systemic administration of GluN2B receptor selective antagonists inhibited behavioral responses to peripheral inflammation. These results are in good accordance with our previous report, showing that GluN2B forebrain overexpression selectively enhanced inflammation-related persistent pain in transgenic mice [15]. Furthermore, we believe that these findings provide critical evidence that NMDAR GluN2B receptors undergo long-term plastic changes in the brain after injury. It should be noted that neurons of the ACC have been implicated in other brain functions, and our present results do not rule out roles for NMDAR GluN2B receptors in other ACC-related physiological functions.

We believe that this GluN2B receptor up-regulation is likely to be reliant on activity-dependent mechanisms. Several lines of evidence support this prediction: (1) the molecular motor protein KIF 17 has been shown to be involved in the active transport of GluN2B [52–54]; (2) GluN2B, mRNA and protein, is highly expressed in ACC neurons [15] and (3) GluN2B contains a CREB (cAMP response element-binding protein) binding domain which may couple increases in intracellular calcium with the increase in GluN2B expression. Since NMDARs play an important role in activity-dependent plasticity in the ACC, we suggest that GluN2B may be regulated through NMDA–calcium–CaM-dependent signaling pathways. The activation of NMDARs trigger postsynaptic calcium, leading to the activation of calcium-stimulated CREB in the ACC after injury [23]. Since GluN2B contains a CREB binding domain, it is likely that GluN2B may be activated downstream from the CREB signaling pathway through the activation of NMDARs. A recent study showed that persistent pain induced by tissue inflammation or nerve injury were significantly reduced in PDZ-93 knockout mice, in part due to the lower level of GluN2B expression at the spinal and cortical levels in knockout mice [55]. Yang et al. reported that Caveolin-1 directly binding with GluN2B subunit and promotion of GluN2B surface levels in the ACC contributed to modulation of chronic neuropathic pain. Disrupting the interaction of Cav-1 and GluN2B through microinjection of a short peptide derived from the C-terminal of GluN2B into the ACC exhibited a significant antinociceptive effect associated with decrease of surface GluN2B expression [56].

Behavioral and pharmacological experiments consistently demonstrate that inhibiting IC LTP produce analgesic effects in animal models of chronic pain. In an animal model of neuropathic pain, microinjection of AMPAR/KAR antagonist 6-cyano-7-nitroquinoxaline-2,3-dione (CNQX) bilaterally into the IC significantly reduced injury related behavioral sensitization. Similar analgesic effects have been found with microinjections of NMDAR antagonist AP5 or a selective GluN2B-NMDARs antagonist Ro 25-6981 bilaterally into the IC [41]. PKMζ activity has been known to be important for maintaining potentiation in different parts of the brain, including the hippocampus and the ACC [57, 58]. Although its role in IC potentiation remain to be examined, microinjection of zeta-pseudosubstrate inhibitory peptide (ZIP), an inhibitor of PKMζ and other PKC isoforms, into the IC produced analgesic effects in rats with nerve injury [59]. Interestingly, the critical roles of IC PKMζ in the conditioned taste aversion (CTA) have been reported. While inhibiting PKMζ produced erasure of long-term memory in the CTA test [60], overexpression of PKMζ in the IC can enhance consolidated long-term memory [61].

## Conclusion and Future Directions

Discovery of the basic mechanism of NMDAR dependent LTP has greatly improved our understanding of brain plasticity. These forms of plasticity not only contribute to various key physiological functions such as learning and memory, but also contribute to the development of pathological conditions that are still difficult to be treated, such as chronic pain and drug addiction. The involvement of NMDAR dependent LTP in pain modulation is better supported by pharmacological inhibitors that are reducing or blocking the expression of LTP without affecting the function of NMDARs such as ZIP.

The complaints of poor translation from animal studies to clinical treatment are not just that these basic mechanisms do not exist in human brains. Instead, poor funding and government regulation have supported less translational works as compared with basic researches. Furthermore, the side effects of various chemicals targeted at NMDARs, including various subtypes, have prevented this translational discovery into human treatment.

One possible solution is selectively targeting downstream proteins from NMDARs, for example, Ca^2+^ stimulation AC1. AC1 serves as a key downstream signaling enzyme in sensory neurons, from spinal dorsal horn to cortical neurons. While genetic deletion of NMDARs caused developmental defects and impairment of cognitive functions, gene deletion of AC1 produce no or minor behavior defects. One possible explanation is that other forms of ACs, as well as downstream protein kinases, may compensate or take over key physiological functions. We hope that fully mapping the signaling pathways of LTP in sensory and cortical neurons may help us to identify new targets, and eventually help to treat patients with different illness such as chronic pain and drug addiction.

## Abbreviations

AC: Adenylyl cyclase
ACC: Anterior cingulate cortex
AMPA: α-Amino-3-hydroxy-5-methyl-4-isoxazole-propionic acid
AP5: d-2-Amino-5-phosphonopentanoic acid
BAPTA: 1,2-Bis(*o*-aminophenoxy)ethane-*N,N,N*′,*N*′-tetraacetic acid
BDNF: Brain-derived neurotrophic factor
CaM: Calmodulin
cAMP: Cyclic AMP (cAMP)
CNQX: 6-Cyano-7-nitroquinoxaline-2,3-dione
CP-AMPARs: Ca^2+^ permeable AMPA receptors
CPP: 3-(R-2-carboxypiperazin-4-yl)-propyl-1-phosphonic acid
CTA: Conditioned taste aversion
EPSCs: Excitatory postsynaptic currents
EPSPs: Excitatory postsynaptic potentials
IC: Insular cortex
KAR: Kainate receptor
LTP: Long-term potentiation
mGluRs: Metabotropic glutamate receptors
NAPSM: 1-Naphthyl acetyl spermine
NKA: Neurokinin A
NMDA: *N*-Methyl-d-aspartate
PDZ: Postsynaptic density-95/Discs large/zona occludens-1
PKC: Protein kinase C
PKMζ: Protein kinase Mζ
PPF: Paired-pulse facilitation
SP: Substance P
ZIP: ζ-Pseudosubstrate inhibitory peptide
